# Perceived Disinformation, Anxiety, and Depressive Symptoms in the Czech Population: Age‐Specific Associations

**DOI:** 10.1155/da/3938343

**Published:** 2026-09-29

**Authors:** Vaclav Moravec, Beata Gavurova, Martin Rigelsky, Michal Miovsky

**Affiliations:** ^1^ Department of Addictology, First Faculty of Medicine, Charles University, Prague, Czech Republic, cuni.cz; ^2^ Faculty of Management and Business, University of Prešov, Prešov, Slovak Republic

**Keywords:** anxiety symptoms, depressive symptoms, digital ecosystem, disinformation, GAD-7, infodemic, PHQ-9, post-COVID period

## Abstract

The main goal of the study was to examine the relationship between anxiety and depressive symptoms and the perception of disinformation in the Czech population. The research was performed on a sample of 2867 respondents. Analytical processing was carried out through quasi‐Poisson regression analysis separately for the three age groups (aged 16–39, 40–64, and 65–90). The dependent variables were the GAD‐7 (Generalised Anxiety Disorder) and PHQ‐9 (Patient Health Questionnaire) total scores, measuring anxiety and depressive symptoms, respectively. The independent variables were represented by the subjective perception of exposure, severity of disinformation, and credibility of specific disinformation in the Czech Republic. The 11 narratives, verified by a fact‐checking organisation, were examined, of which eight were disinformation claims and three were factual reports. The outcomes demonstrated that 69.3% of the population experienced no or minimal anxiety, 19.0% mild, 7.2% moderate, and 4.5% severe anxiety. In the case of depression, 43.0% of the population demonstrated minimal symptoms, 25.4% mild, 9.9% moderate, and 14.3% no symptoms. After Benjamini–Hochberg false‐discovery‐rate (FDR) adjustment, perceived exposure to disinformation remained positively associated with GAD‐7 and PHQ‐9 scores across all three age groups. Among the 66 narrative‐specific tests, 20 of 33 GAD‐7 associations and 17 of 33 PHQ‐9 associations remained statistically significant after FDR correction. The retained associations varied by narrative and age group rather than indicating a uniform age gradient. Because the data are cross‐sectional, these associations do not establish causal direction.

## 1. Introduction

During crises involving outbreaks of infectious disease, disasters, or armed conflicts, the rapid production and dissemination of information from multiple sources, together with varying information quality, can have serious social and public health consequences. The flood of online information containing either true or misleading information, referred to as infodemics [1], has created pressures to understand the general impact of infodemics on society, reveal patterns of spread, and define appropriate countermeasure policies. Many studies analyse the societal and public health effects of infodemics and disinformation [2–6], but fewer directly examine their associations with specific anxiety and depressive symptoms.

Social media are among the fastest and most accessible sources of information for a large part of the population, and they have substantially changed how people obtain and discuss crisis‐related information [7–10]. Concurrently with this development, anxiety and depressive symptoms have become a major concern in some population groups, especially among young people, and concerns have arisen that the use of social media threatens mental health [7, 8]. Anxiety and depressive symptoms in online environments may be associated with trust, perceived safety, uncertainty, and the time and cognitive energy devoted to information seeking [9]. Prasad et al. [10] reported that specific patterns of social media engagement are associated with anxiety and depression among young people. More detailed research is required to determine directionality and causality because anxiety and depressive symptoms may be associated with threatening information environments but may also shape attention to and interpretation of such environments.

The COVID‐19 pandemic provided a particularly salient context in which the broader phenomenon of an infodemic became closely intertwined with the rapid circulation of disinformation, heightened uncertainty, and concerns about anxiety and depressive symptoms [11, 12]. During the pandemic, social media were saturated with conspiracy theories and disinformation, especially concerning vaccination; exposure to this content was associated with greater uncertainty, anxiety, depressive symptoms, panic, and behavioural changes [13, 14].

Belief in conspiracy theories and disinformation varies across geographical regions and sociodemographic groups, which may also differ in mental health vulnerability and patterns of media use. For instance, Conninck et al. [15] found that the United States, the Philippines, and Hong Kong ranked in the top three for belief in conspiracy theories and disinformation, whereas Canada, Switzerland, Belgium, and New Zealand, whose populations appeared more resilient to disinformation, achieved lower scores. While traditional media consumption—related to television, radio, and newspapers—demonstrates an inverse relationship with conspiracy endorsement and susceptibility to disinformation, exposure to digital media, political figures, and personal networks positively correlates with these beliefs. These findings are directly relevant to the current investigation, which explores how sociodemographic variables and media usage intersect with empirical risk for anxiety and depressive symptoms.

According to the study by Conninck et al. [15], age is a significant predictor of belief in disinformation and conspiracy theories, with younger respondents believing more disinformation narratives than older people. Older age was associated with greater resilience to disinformation in that study. Sex was confirmed as a significant predictor for belief in disinformation but not for conspiracy theories. Belief in conspiracy theories was influenced by educational attainment—more educated people showed weaker belief in conspiracy theories about COVID‐19. The level of trust in disinformation narratives was less influenced by educational level and media literacy, which requires a deeper examination of the reasons. Jabbour et al. [16] examined the impact of social media on the perception of disinformation and on students’ mental health and their decisions and reported a low frequency of mental health disorders among university students that was associated with frequent use of social media during the COVID‐19 pandemic. Their findings highlight the importance of promoting healthier patterns of social media use and examining how these patterns relate to mental health and decision‐making at both individual and community levels. Prisă [17] provided convincing evidence on the role of a person’s cognitive profile, locus of control, and intolerance of uncertainty on emotional states induced by media information, especially fake news. The study also confirmed the differences in dysfunctional negative emotional states induced by disinformation depending on age (higher values of anxiety and anger in the group of the population aged over 35 compared to younger categories). These findings suggest that cognitive profile and intolerance of uncertainty may influence both responses to disinformation and symptoms of anxiety or depression.

According to some studies, belief in disinformation and conspiracy theories is more often negatively associated with the use of traditional media and positively associated with digital media and personal contacts. Conninck et al. [15] point out the inconsistency in the effects of trust in politics on belief in disinformation across some research studies (for instance, [18]) and confirm that trust in politics acts as a catalyst for belief in disinformation. Exposure to a large amount of information in a short time period can promote the generation of hypotheses, speculations, and conspiracy theories (for instance, [19, 20]), as well as excessive use of and trust in digital media and personal contacts. Conninck et al. [15] confirm that anxiety is not strongly associated with beliefs in conspiracies or disinformation in most of the examined countries, whereas depression was associated with higher levels of belief in disinformation and conspiracy theories.

Jobson et al. [21] examined the relationships between the disinformation effect, trauma exposure, post‐traumatic stress disorder (PTSD), and depression. The authors found that depression and PTSD involve automatic and rapid activation of associative networks when individuals encounter emotional stimuli and experiences consistent with their disorders, suggesting that vulnerability to potential disinformation effects may vary according to mental health status. According to some studies, the form of presentation of disinformation also has an impact on its effects. Lee et al. [22] investigated whether disinformation presented through image or video formats can be more powerful than disinformation presented in text‐only format. Anxiety may be an important mechanism linking exposure to disinformation with individual misperceptions. Higher PTSD symptom levels have also been associated with a greater likelihood of believing false news, with fear of missing out (FOMO) proposed as a possible explanatory mechanism [23].

Disinformation and conspiracy theories are not only associated with the COVID‐19 pandemic, but their emergence and spread have also continued in other types of crises and geopolitical conflicts. Vintilă et al. [24] examined the impact of social media consumption and excessive information related to the conflict between Russia and Ukraine on the spread of fake news within the Romanian population. Some psychological characteristics, including resilience, general health, perceived stress and coping strategies, and fear of war, may vary depending on individuals’ exposure to past traumatic events or interactions with war victims [25]. Vintilă et al. [24] suggest that informational pressure partially moderates the link between information overload (quantified as time spent online) and the propensity to disseminate disinformation. Synthesising these findings with broader empirical literature, it is plausible to hypothesise that individuals with high social media exposure who experience elevated information overload and crisis‐related distress are significantly more inclined to propagate unverified information rather than engage in systematic fact‐checking or critical filtering [26, 27]. Exposure to disinformation has also been associated with anxiety and social media fatigue among employees working from home, which in turn were linked to lower work engagement and performance [28].

The literature examining relationships between perceived disinformation and depression or anxiety remains heavily concentrated on the COVID‐19 and post‐pandemic periods and is therefore dominated by health‐related disinformation. Disinformation circulated through social media extends into everyday life [29, 30] and is not limited to crisis or pandemic events. It encompasses national and international security, environmental and health issues, social policy, geopolitical developments, political stability, and social crises. The research studies investigating the relationships between the perception of current disinformation with different thematic focuses and their associations with anxiety and depressive symptoms remain limited. The reason may also be the considerable methodological difficulty, which includes the accessibility of current and verified disinformation requiring cooperation with fact‐checking institutions belong among—fact‐checking organizations, national news agencies, and so on [31], as well as understanding the seriousness of the impacts of various types of disinformation for the individual and society [32].

These facts justify the research gap that this study focuses on. Although the acute phase of the COVID‐19 pandemic has passed, the broader challenge of infodemics persists, with false information often spreading farther and faster than accurate information [33]. Stress and uncertainty may encourage repeated information seeking intended to reduce anxiety or restore a sense of control, while simultaneously increasing exposure to further alarming or contradictory content [17, 26–28].

A bidirectional informational‐stress and cognitive‐vulnerability framework helps to organise these relationships. Perceived exposure to and seriousness of disinformation may increase uncertainty, information overload, perceived threat, and repetitive information seeking. These processes may be relevant to GAD‐7 (generalised anxiety disorder) anxiety symptoms through persistent worry, nervousness, restlessness, and heightened threat vigilance and to PHQ‐9 (Patient Health Questionnaire) depressive symptoms through rumination, perceived helplessness, reduced interest, fatigue, concentration difficulties, and negative interpretation [17, 21, 26–28, 34–37]. In the opposite direction, symptoms of anxiety are likely to increase threat sensitivity and impede detachment from distressing information, while depressive symptoms intensify rumination, negative attentional bias, and the endorsement of mood‐congruent narratives. Consequently, affective distress may actively drive engagement with and propagation of alarming content [21, 37]. Analytical thinking focused on accuracy may protect against disinformation [27, 38], although anxiety, distraction, and cognitive overload may reduce the likelihood that it is applied. Shared factors, including education, socioeconomic status, media use, institutional trust, and prior mental health, may also influence both disinformation perceptions and symptom scores. The cross‐sectional design of the present study can identify associations but cannot determine their temporal or causal direction.

Accordingly, the aim of the study was to examine associations between perceptions of current disinformation from different thematic fields and GAD‐7 anxiety symptom scores and PHQ‐9 depressive symptom scores in the Czech population.

To achieve the aim of the study, the following research questions (RQs) were set:

RQ1: Are perceived exposure to and seriousness of disinformation associated with GAD‐7 anxiety‐symptom and PHQ‐9 depressive‐symptom scores among Czech respondents?

RQ2: Is the perceived credibility of individual information narratives associated with GAD‐7 and PHQ‐9 scores among Czech respondents?

## 2. Materials and Methods

### 2.1. Data Source and Study Population

The research was carried out in the Czech Republic as a part of the CEDMO Trends project—the Czech society in the period of change.

Respondents were selected from a pre‐established panel, which was created prior to the launch of the survey on the basis of the MEDIAN online panel’s respondent database. This panel was compiled in accordance with guidelines approved by the client commissioning the survey. The research panel was adjusted to represent the general population of the Czech Republic, taking into account the characteristics specified by the research client.

Data collection took place between 10 January 2025 and 26 January 2025 through the research agency MEDIAN s.r.o. The research sample included the population aged over 16 and quota selection was carried out. The monitored quotas were: sex, age, education, region, size of place of residence, Internet use, and voting behaviour in the 2021 parliamentary elections and the 2023 presidential elections.

A structured questionnaire was used as a part of the CEDMO study. Each respondent was sent an invitation to participate in the survey by email. All participants provided written informed consent to their inclusion in the study prior to taking part. Once data collection had commenced, no further modifications were made to the questionnaire.

The total number of respondents approached was 4198, of whom 1073 respondents did not respond to the invitation to complete the questionnaire (i.e., a response rate of 74.4%), 44 respondents who began filling in the questionnaire were excluded on the basis of a filter (attention check), and 108 respondents did not complete the questionnaire. Of the 2973 respondents who completed the entire questionnaire, 106 questionnaires were excluded during data quality checks (in 5 cases, the completion time was less than a quarter of the median; in 5 cases, the reason was inconsistent answers; and in 96 cases, there were missing data in the questionnaire). A total of 2867 questionnaires were included in the analytical processes. The final analytical sample therefore represented 68.3% of all invited panel members and 91.7% of those who responded to the invitation and entered the survey process.

Each respondent was given at least 3 days to complete the questionnaire. Approximately one‐third of respondents received at least one reminder to complete it. Respondents were offered a reward for completing the questionnaire in line with MEDIAN’s standard remuneration rates. 13% of respondents donated their reward to a charity. The average time taken to complete the questionnaire was 26.5 min. Comparable sociodemographic information was not available for all nonrespondents, incomplete cases, and records removed during data quality control; therefore, demographic selection bias between the 2867 analysed cases and the entire excluded group could not be quantified directly.

The study was conducted in accordance with the Declaration of Helsinki, and the protocol was approved by the Ethics Committee of the General University Hospital in Prague (IORG0002175—General University Hospital in Prague, IRB00002705, and Federalwide Assurance FWA00029052).

Table 1 contains the basic characteristics of the final analytical sample. Quota controls were used to approximate the target Czech population on sex, age, education, region, size of place of residence, Internet use, and previous voting behaviour; however, the CAWI panel design, nonresponse, and quality‐control exclusions mean that the final sample should not be interpreted as a probability sample and may retain residual selection bias. Sex was almost evenly represented (48.1% men and 51.9% women). Respondents aged 65 years and older represented 30.6% of the sample. The largest educational groups were respondents with secondary education without a school‐leaving diploma (35.6%) and with a school‐leaving diploma (36.8%). The smallest residence‐size category was large cities (23.5%). Employees represented 41.5% and nonworking pensioners 30.6% of the sample. The sample was digitally active, with 71.3% reporting Internet use several times a day.

**Table 1:** Research sample characteristics.

Variable	Frequency	Percentage
Sex		
Male	1378	48.1
Female	1489	51.9
Age: six categories		
16–24	165	5.8
25–34	252	8.8
35–44	462	16.1
45–54	605	21.1
55–64	505	17.6
65+	878	30.6
Education: four categories		
Primary	213	7.4
Secondary	1020	35.6
Secondary with a diploma	1056	36.8
Tertiary	578	20.2
Residence size: three categories		
Up to 4999 inhabitants	1056	36.8
5000–99,999 inhabitants	1136	39.6
Over 100,000 inhabitants	675	23.5
Economic status		
Employees (including working pensioners)	1191	41.5
Entrepreneurs	292	10.2
Unemployed	90	3.1
Pensioners (nonworking)	876	30.6
Students (working and nonworking)	138	4.8
Others (on maternity leave, in household, other)	280	9.8
Internet frequency use		
Less often	212	7.4
Once a day or almost daily	611	21.3
Several times a day	2044	71.3

Figure 1 presents the distribution of respondents across three age groups: 16–39 years (*n* = 628, 21.9%), 40–64 years (*n* = 1361, 47.5%), and 65 years and older (*n* = 878, 30.6%). These groups were defined to reflect broad life course stages and to facilitate the examination of potential age‐related differences in anxiety and depressive symptoms, perceived exposure to disinformation, and the perceived credibility of individual information narratives.

**Figure 1:**
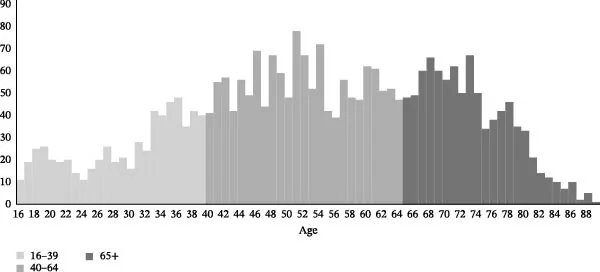
Distribution of respondents by age and age groups.

Separate analyses were conducted for each age group because preliminary Kruskal–Wallis tests identified statistically significant differences between age categories for both mental health outcomes and for most variables related to disinformation perception. This stratified approach was therefore used to describe and explore age‐specific patterns in the associations between perceptions of disinformation and anxiety or depressive symptom scores. Although stratification reduces the sample size available for individual models, the group sizes remained sufficient for the planned analyses, and this approach enabled a more transparent comparison of patterns across the three age categories.

### 2.2. Study Measures

The analyses applied GAD‐7 and PHQ‐9 as the dependent variables. The standard methodology of Patient Health Questionnaire PHQ‐9 [34, 35] and GAD‐7 [35, 36] was applied for the survey. Both instruments (GAD‐7 and PHQ‐9) were translated from English to Czech by Pfizer (Pfizer Inc., New York City, NY, USA), and the translated versions were used in this study [39].

The GAD‐7 instrument consisted of the seven items: Feeling nervous, anxious, or on edge (GAD‐7A); not being able to stop or control worrying (GAD‐7B); worrying too much about different things (GAD‐7C); trouble relaxing (GAD‐7D); being so restless that it is hard to sit still (GAD‐7E); becoming easily annoyed or irritable (GAD‐7F); and feeling afraid, as if something awful might happen (GAD‐7G). The items were measured on a four‐point scale (Not at all—0, Several days—1, More than half the days—2, Nearly every day—3).

Table 2 demonstrates the frequencies and percentages of responses on the GAD‐7 scale for individual items (GAD‐7A–GAD‐7G). The columns represent the categories of anxiety symptoms: “Not at all”, “Several days”, “More than half the days”, and “Nearly every day”. Each row shows the number of respondents and their percentage in the respective category for a given GAD‐7 statement. After processing the GAD‐7 scale, it was found that the average value in the Czech Republic is 3.72 (median = 2, SD = 4.65, min = 0, and max = 21). Based on this raw score, respondents were classified into four levels of severity of anxiety symptoms, with none, or 1986 (69.3%) respondents experience minimal problems, 546 (19.0%) at the mild level, 206 (7.2%) at the moderate level, and 129 (4.5%) at the most intense anxiety level. The severity of generalised anxiety symptoms was interpreted according to the total GAD‐7 score: scores of 0–4 indicated minimal anxiety, scores of 5–9 mild anxiety, scores of 10–14 moderate anxiety, and scores of 15–21 severe anxiety. Both item development and scoring were based on the study by Spitzer et al. [36].

**Table 2:** Frequency analysis—GAD‐7.

Item	Not at all	Several days	More than half the days	Nearly every day
GAD‐7A	1700 (59.3%)	762 (26.58%)	223 (7.78%)	182 (6.35%)
GAD‐7B	2013 (70.21%)	542 (18.9%)	194 (6.77%)	118 (4.12%)
GAD‐7C	1457 (50.82%)	984 (34.32%)	227 (7.92%)	199 (6.94%)
GAD‐7D	1668 (58.18%)	821 (28.64%)	201 (7.01%)	177 (6.17%)
GAD‐7E	2240 (78.13%)	406 (14.16%)	132 (4.6%)	89 (3.1%)
GAD‐7F	1608 (56.09%)	884 (30.83%)	236 (8.23%)	139 (4.85%)
GAD‐7G	2082 (72.62%)	552 (19.25%)	119 (4.15%)	114 (3.98%)

The PHQ‐9 instrument consisted of nine items: Little interest or pleasure in doing things (PHQ‐9A); feeling down, depressed, or hopeless (PHQ‐9B); trouble falling or staying asleep, or sleeping too much (PHQ‐9C); feeling tired or having little energy (PHQ‐9D); poor appetite or overeating (PHQ‐9E); feeling bad about yourself or that you are a failure or have let yourself or your family down (PHQ‐9F); trouble concentrating on things, such as reading the newspaper or watching television (PHQ‐9G); moving or speaking so slowly that other people could have noticed. Or the opposite being so fidgety or restless that you have been moving around a lot more than usual (PHQ‐9H); thoughts that you would be better off dead, or of hurting yourself (PHQ‐9I). The items were investigated on a four‐point scale (Not at all—0, Several days—1, More than half the days—2, Nearly every day—3).

Table 3 presents the frequencies and percentages of responses on the PHQ‐9 scale for individual items (PHQ‐9A–PHQ‐9I). The columns represent the frequency of occurrence of depressive symptoms in individual categories: Not at all, Several days, More than half the days, and Nearly every day. Each row contains the number of respondents and their percentage representation in a given category for the corresponding PHQ‐9 scale item. The average PHQ‐9 score in the analysed sample was 5.23 (median = 4, SD = 5.19, min = 0, and max = 27). Based on this score, respondents were classified into six categories according to the severity of depressive symptoms: minimal (1234 respondents, 43.0%), mild (727 respondents, 25.4%), moderate (284 respondents, 9.9%), moderately severe (148 respondents, 5.2%), and severe (64 respondents, 2.2%), with 410 respondents (14.3%) reporting no depressive symptoms. The severity of depressive symptoms was interpreted according to the total PHQ‐9 score: a score of 0 indicated no depressive symptoms, scores of 1–4 indicated minimal depressive symptoms, scores of 5–9 mild depression, scores of 10–14 moderate depression, scores of 15–19 moderately severe depression, and scores of 20–27 severe depression. Both item development and scoring were based on the study by Kroenke et al. [34].

**Table 3:** Frequency analysis—PHQ‐9.

Item	Not at all	Several days	More than half the days	Nearly every day
PHQ‐9A	1601 (55.84%)	866 (30.21%)	256 (8.93%)	144 (5.02%)
PHQ‐9B	1993 (69.52%)	571 (19.92%)	185 (6.45%)	118 (4.12%)
PHQ‐9C	1071 (37.36%)	1070 (37.32%)	336 (11.72%)	390 (13.6%)
PHQ‐9D	710 (24.76%)	1350 (47.09%)	421 (14.68%)	386 (13.46%)
PHQ‐9E	1815 (63.31%)	697 (24.31%)	180 (6.28%)	175 (6.10%)
PHQ‐9F	1850 (64.53%)	692 (24.14%)	189 (6.59%)	136 (4.74%)
PHQ‐9G	1940 (67.67%)	668 (23.30%)	169 (5.89%)	90 (3.14%)
PHQ‐9H	2426 (84.62%)	289 (10.08%)	112 (3.91%)	40 (1.40%)
PHQ‐9I	2421 (84.44%)	285 (9.94%)	100 (3.49%)	61 (2.13%)

IP02, IP04, and DEZ represented the independent variables.

IP02 (To what extent in the past month, in your opinion, were you or were you not exposed to purposefully disseminated false or manipulative information (sometimes also referred to as “disinformation”) in the media?) was measured on a four‐point scale: Very exposed (4)—105 (3.7%), More exposed (3)—420 (14.7%), More often not exposed (2)—1226 (42.7%), and Not exposed at all (1)—1116 (38.9%).

IP04 (How serious a problem do you think the spread of false and manipulative information (sometimes also referred to as “disinformation”) is on the Internet?) presented the level of perception of the seriousness of the spread of false and manipulative information on the Internet and was assessed on a five‐point scale: Very serious problem (4)—888 (31.4%), Quite serious problem (3)—1066 (37.2%), Slightly serious problem (2)—512 (17.9%), Not a problem at all (1)—176 (6.2%), and I do not know/cannot judge (NA)—211 (7.4%).

The particular items entered the analytical processing—specific narratives measuring subjective credibility were also included in the analytical process. The items were evaluated on a four‐point scale, where Completely Unbelievable was coded as 1 (the lowest possible numerical value) and Completely Believable as 4 (the highest numerical value of credibility).

In quantitative research, the Czech population was tested on the eight most often spread disinformation narratives that were caught by the fact‐checking organisations of the CEDMO research hub. The listed narratives were spread through social networks in November and December 2024. The sample of the eight disinformation narratives from the various thematic fields was supplied by the three true narratives (DEZ01I, DEZ01J, and DEZ01K) published by the Czech News Agency as a public service medium. Altogether, the 11 narratives were tested as a part of the quantitative investigation regarding awareness and their credibility. The narratives and frequency analysis are listed below:

• DEZ01A. Bethlehem Square was officially renamed Náměstí Volodymyr Zelenský in Prague, which bears the name of the current president of Ukraine: Totally incredible 1650 (57.6%), Rather incredible 897 (31.3%), Rather credible 252 (8.8%), and Completely credible 68 (2.4%).
• DEZ01B. The COVID‐19 pandemic was a targeted military operation of the North Atlantic Alliance (NATO), which was recently confirmed by the Dutch Minister of Health, Fleur Agema: Completely incredible 1363 (47.5%), Rather 991 (34.6%), Rather credible 390 (13.6%), and Completely credible 123 (4.3%).
• DEZ01C. The government of Petr Fiala wants to burden not only them themselves but also their family members with mandatory payments for the care of pensioners in homes for the elderly. In addition, payments will be calculated according to the properties owned by relatives of seniors: Completely incredible 781 (27.2%), Rather incredible 1113 (38.8%), Rather credible 740 (25.8%), and Completely credible 233 (8.1%).
• DEZ01D. Butter prices in Europe, including the Czech Republic, due to the lack of milk fat, reduce again the fat from insect larvae. In the future, insect products should make up to a quarter of the weight of butter: Completely incredible 1431 (49.9%), Rather incredible 1075 (37.5%), Rather credible 300 (10.5%), and Completely credible 61 (2.1%).
• DEZ01E. The Swedish government has passed a resolution that the science of man‐made global warming is a hoax. In addition, Sweden backed away from its commitment to become a climate‐neutral state by 2045: Completely incredible 875 (30.5%), Rather incredible 1385 (48.3%), Rather credible 517 (18.0%), and Completely credible 90 (3.1%).
• DEZ01F. As part of an important step in the brewing industry in Central Europe, the popular Czech brand Braník was purchased by the leading Ukrainian brewing company Obolon: Totally incredible 766 (26.7%), Rather incredible 1510 (52.7%), Rather credible 509 (17.8%), and Completely credible 82 (2.9%).
• DEZ01G. During the Czech Presidency in 2022, the European Union approved a climate tax on real estate. Its consequence is the expropriation of energy‐intensive real estate and an increase in homelessness: Completely incredible 1057 (36.9%), Rather incredible 1200 (41.9%), Rather credible 496 (17.3%), and Completely credible 114 (4.0%).
• DEZ01H. Most of the arable land in Ukraine has been sold to foreign owners in the past 4 years. The rest of the arable land is owned by Ukrainian oligarchs: Totally incredible 604 (21.1%), Rather incredible 1313 (45.8%), Rather credible 772 (26.9%), and Completely credible 178 (6.2%).
• DEZ01I. In spite of his promises, the incumbent US President Joe Biden pardoned his son Hunter in cases of illegal acquisition of weapons and tax offences: Totally incredible 132 (4.6%), Rather incredible 570 (19.9%), Rather credible 1157 (40.4%), and Completely credible 1008 (35.2%).
• DEZ01J. Roughly 1.2 million people in the Czech Republic have been paying lower amounts for prescription drugs in pharmacies since January, which is related to the change in the administration of the protective limit. This mostly concerns seniors over 70 who regularly take several medications: Completely incredible 194 (6.8%), Rather incredible 690 (24.1%), Rather credible 1440 (50.2%), and Completely credible 543 (18.9%).
• DEZ01K. Bashar Assad was transported to Russia in a very safe manner. The Kremlin said on Monday that Russian President Vladimir Putin has decided to grant asylum to Assad: Totally incredible 173 (6.0%), Rather incredible 574 (20.0%), Rather credible 1448 (50.5%), and Completely credible 672 (23.4%).

Eight items (DEZ01A–DEZ01H) were verified disinformation claims, whereas three items (DEZ01I–DEZ01K) were verified factual reports. All items were analysed separately as information narratives to examine associations between perceived credibility and anxiety and depressive symptom scores.

The narratives were analysed separately because they represented specific, thematically diverse, and time‐bound claims related to distinct social, political, health, environmental, and geopolitical events. Treating the narratives as separate predictors preserved their specific informational value and enabled the identification of distinct associations between the perceived credibility of individual claims and mental health outcomes.

The narrative items were not designed or psychometrically validated as a scale of general susceptibility to disinformation. They operationalised perceived credibility of specific, contemporaneous claims whose factual status had been independently established. Credibility ratings may therefore reflect topic‐specific knowledge, prior exposure, political or social attitudes, familiarity with the issue, and other individual predispositions in addition to the evaluation of misleading information. Respondents evaluated brief statements without the original source, surrounding discourse, visual presentation, or other contextual credibility cues. The task therefore did not constitute a performance‐based assessment of source evaluation, lateral reading, contextual fact‐checking, or general media literacy [40].

### 2.3. Statistical Analysis

In the first step of the analytical processes, analysis of variance was applied, where the differences in the selected indicators between age groups were identified through the Kruskal–Wallis non‐parametric test. Arithmetic mean and median were applied to identify central tendencies. Subsequently, regression analysis was applied to identify associations between anxiety and depressive symptom scores and perceptions of disinformation. Quasi‐Poisson regression models [41] were used to examine the associations between perceptions of disinformation and GAD‐7 and PHQ‐9 total scores. This approach was considered appropriate because the outcomes are non‐negative discrete scores, and the quasi‐Poisson specification does not impose equality between the conditional mean and variance. This model provides more reliable estimates of standard errors and *p*‐values, thereby reducing the risk of erroneous conclusions. Perceived exposure to disinformation (IP02) and perceived seriousness of online disinformation (IP04) were entered simultaneously into multivariable quasi‐Poisson regression models. Given the number and thematic specificity of the DEZ narratives, each narrative was subsequently examined in a separate univariable quasi‐Poisson regression model in relation to GAD‐7 and PHQ‐9 total scores. The analytical processes were implemented in the programming language R (v. 4.4.2 Pile of Leaves) and Tableau (v. 2024.3). In the programming environment R, the packages like dplyr [42], tidyr [43], car [44], haven [45], psych [46], MASS [47], purrr [48], and broom [49] were used.

Multiple testing was handled using separate analytical families reflecting distinct purposes. The 15 preliminary Kruskal–Wallis age‐group comparisons reported in Table 4 were treated as a screening family and adjusted using the Benjamini–Hochberg false‐discovery‐rate (FDR) procedure. For RQ1, the family comprised 12 coefficient tests (IP02 and IP04 across two outcomes and three age groups). For RQ2, the family comprised 66 narrative‐specific tests (11 narratives across two outcomes and three age groups). Statistical interpretation was based on FDR‐adjusted *q*‐values, with *q* < 0.05 considered statistically significant. Intercepts were not included in either regression correction family. Because some archived outputs recorded very small *p*‐values only as *p* < 0.001, a value of 0.001 was used for those entries in the FDR recalculation; the corresponding *q*‐values are therefore conservative.

**Table 4:** Analysis of the differences in age group classification.

Variable	Mean (SD)|Median (IQR)			KW statistic	*p*/*q*
	16–39	40–64	65–90		
PHQ9 score	6.68 (5.55)|5.00 (8.00)	5.29 (5.37)|4.00 (7.00)	4.12 (4.33)|3.00 (5.00)	97.9	<0.001/0.0014
GAD7 score	5.35 (5.05)|4.00 (7.00)	3.72 (4.66)|2.00 (6.00)	2.56 (3.94)|1.00 (3.75)	181.0	<0.001/0.0014
IP02	1.80 (0.76)|2.00 (1.00)	1.87 (0.87)|2.00 (1.00)	1.94 (0.81)|2.00 (1.00)	11.9	0.003/0.0038
IP04	3.19 (0.80)|3.00 (1.00)	3.02 (0.92)|3.00 (2.00)	2.87 (0.89)|3.00 (2.00)	44.7	<0.001/0.0014
DEZ01A	1.47 (0.69)|1.00 (1.00)	1.54 (0.75)|1.00 (1.00)	1.66 (0.78)|1.00 (1.00)	31.2	<0.001/0.0014
DEZ01B	1.60 (0.81)|1.00 (1.00)	1.79 (0.88)|2.00 (1.00)	1.78 (0.82)|2.00 (1.00)	31.0	<0.001/0.0014
DEZ01C	1.93 (0.86)|2.00 (1.00)	2.17 (0.94)|2.00 (2.00)	2.27 (0.89)|2.00 (1.00)	55.1	<0.001/0.0014
DEZ01D	1.62 (0.75)|1.00 (1.00)	1.66 (0.77)|1.00 (1.00)	1.65 (0.72)|2.00 (1.00)	2.2	0.333/0.3330
DEZ01E	1.79 (0.80)|2.00 (1.00)	1.96 (0.79)|2.00 (1.00)	2.02 (0.73)|2.00 (0.00)	43.4	<0.001/0.0014
DEZ01F	1.89 (0.73)|2.00 (1.00)	1.94 (0.75)|2.00 (1.00)	2.07 (0.75)|2.00 (0.00)	26.5	<0.001/0.0014
DEZ01G	1.86 (0.82)|2.00 (1.00)	1.87 (0.84)|2.00 (1.00)	1.92 (0.83)|2.00 (1.00)	2.9	0.238/0.2550
DEZ01H	2.04 (0.76)|2.00 (1.25)	2.16 (0.83)|2.00 (1.00)	2.33 (0.86)|2.00 (1.00)	43.6	<0.001/0.0014
DEZ01I	2.85 (0.89)|3.00 (2.00)	3.02 (0.86)|3.00 (2.00)	3.27 (0.78)|3.00 (1.00)	89.1	<0.001/0.0014
DEZ01J	2.81 (0.75)|3.00 (1.00)	2.78 (0.82)|3.00 (1.00)	2.87 (0.86)|3.00 (1.75)	5.8	0.055/0.0635
DEZ01K	2.67 (0.86)|3.00 (1.00)	2.91 (0.83)|3.00 (1.00)	3.09 (0.71)|3.00 (1.00)	94.0	<0.001/0.0014

## 3. Results

Table 4 presents descriptive statistics for the three age categories, reported as mean (SD) and median (IQR), together with the results of the Kruskal–Wallis tests. For the psychological scales, both PHQ‐9 and GAD‐7 scores decreased consistently with age, with the youngest respondents reported the highest levels of depressive symptoms (*M* = 6.68, SD = 5.55; Md = 5.00, IQR = 8.00) and anxiety symptoms (*M* = 5.35, SD = 5.05; Md = 4.00, IQR = 7.00), whereas the oldest group reported the lowest scores (PHQ‐9: *M* = 4.12, SD = 4.33; Md = 3.00, IQR = 5.00; GAD‐7: *M* = 2.56, SD = 3.94; Md = 1.00, IQR = 3.75). Differences between age groups were statistically significant for both scales (PHQ‐9: *H* = 97.9, *p* < 0.001; GAD‐7: *H* = 181.0, *p* < 0.001). Statistically significant age‐group differences were also observed for IP02, IP04, and most DEZ items, specifically DEZ01A, DEZ01B, DEZ01C, DEZ01E, DEZ01F, DEZ01H, DEZ01I, and DEZ01K (all *p* < 0.01). In contrast, no statistically significant differences were found for DEZ01D (*H* = 2.2, *p* = 0.333), DEZ01G (*H* = 2.9, *p* = 0.238), or DEZ01J (*H* = 5.8, *p* = 0.055). Overall, the findings indicate that age is associated with substantial differences in depressive and anxiety symptomatology as well as in the majority of the examined variables. Therefore, conducting the subsequent regression analyses separately for individual age groups is justified as it allows potential age‐specific patterns in the relationships among the variables to be identified. After Benjamini–Hochberg correction across the 15 Kruskal–Wallis comparisons, the inferential pattern was unchanged: PHQ‐9, GAD‐7, IP02, IP04, DEZ01A, DEZ01B, DEZ01C, DEZ01E, DEZ01F, DEZ01H, DEZ01I, and DEZ01K remained significant, whereas DEZ01D, DEZ01G, and DEZ01J did not (Table 4).

### 3.1. RQ1: Are Perceived Exposure to and Seriousness of Disinformation Associated With GAD‐7 Anxiety‐Symptom and PHQ‐9 Depressive‐Symptom Scores Among Czech Respondents?

The outcomes of the analytical processes in Tables 5 and 6 are linked to RQ1.

**Table 5:** Multiple quasi‐Poisson regression analysis—linking attitudes to disinformation with GAD‐7 (score) in age group classification.

Term	Aged 16–39			Aged 40–64			Aged 65–90		
	*β* (95% CI)	*p*‐Value *q*	*R* ^2^	*β* (95% CI)	*p*‐Value *q*	*R* ^2^	*β* (95% CI)	*p*‐Value *q*	*R* ^2^
*α*	1.01 (0.653–1.37)	<0.001 —	0.125|0.457	0.288 (0.002–0.575)	0.049 —	0.128|0.501	0.143 (−0.303–0.59)	0.529 —	0.11|0.421
IP02	0.159 (0.065–0.254)	0.001 0.0017		0.180 (0.106–0.254)	<0.001 0.0017		0.238 (0.114–0.362)	<0.001 0.0017	
IP04	0.108 (0.009–0.207)	0.032 0.0427		0.210 (0.13–0.291)	<0.001 0.0017		0.096 (−0.024–0.217)	0.115 0.1380	

*Note: R* ^2^: McFadden|Nagelkerke. FDR *q*‐values are Benjamini–Hochberg adjusted within the 12‐test RQ1 family; intercepts were not included in the correction.

**Table 6:** Multiple quasi‐Poisson regression analysis—linking attitudes to disinformation with PHQ‐9 (score) in age group classification.

Term	Aged 16–39			Aged 40–64			Aged 65–90		
	*β* (95% CI)	*p*‐Value *q*	*R* ^2^	*β* (95% CI)	*p*‐Value *q*	*R* ^2^	*β* (95% CI)	*p*‐Value *q*	*R* ^2^
*α*	1.57 (1.27–1.88)	<0.001 —	0.096|0.361	0.989 (0.765–1.21)	<0.001 —	0.127|0.482	0.95 (0.654–1.25)	<0.001 —	0.105|0.353
IP02	0.120 (0.034–0.205)	0.006 0.0090		0.133 (0.072–0.194)	<0.0013 0.0017		0.148 (0.063–0.233)	0.001 0.0017	
IP04	0.028 (−0.059–0.114)	0.530 0.5300		0.132 (0.068–0.195)	<0.001 0.0017		0.052 (−0.029–0.132)	0.209 0.2280	

*Note: R* ^2^: McFadden|Nagelkerke. FDR *q*‐values are Benjamini–Hochberg adjusted within the 12‐test RQ1 family; intercepts were not included in the correction.

Table 5 presents the outcomes of a multiple quasi‐Poisson regression analysis that examined the relationship between GAD‐7 anxiety symptom scores and attitudes towards disinformation for three age groups. Multicollinearity was assessed first, and the VIF value did not exceed 5 in any model. After FDR adjustment across the 12 RQ1 coefficient tests, perceived exposure to disinformation (IP02) remained positively associated with GAD‐7 scores in all three age groups (*q* = 0.0017 in each group). The largest coefficient was observed in the 65–90 group (*β* = 0.238). Perceived seriousness of online disinformation (IP04) remained associated with GAD‐7 scores in the 16–39 group (*q* = 0.0427) and the 40–64 group (*q* = 0.0017) but not in the 65–90 group (*q* = 0.1380). These results support an association between disinformation‐related perceptions and anxiety symptoms while showing that the pattern differs by age and by the specific perception measure. The cross‐sectional models do not establish causal direction.

Table 6 presents the outcomes of the multiple quasi‐Poisson regression analysis with PHQ‐9 depressive symptom scores as the dependent variable. Multicollinearity was assessed first, and the VIF value did not exceed 5 in any model. After FDR adjustment, perceived exposure to disinformation (IP02) remained positively associated with PHQ‐9 scores in all three age groups (*q* = 0.0090 for ages 16–39 and *q* = 0.0017 for ages 40–64 and 65–90). Perceived seriousness of online disinformation (IP04) remained associated with PHQ‐9 only in the 40–64 group (*q* = 0.0017); the associations were not significant after correction in the 16–39 (*q* = 0.5300) or 65–90 (*q* = 0.2280) groups. The results therefore indicate age‐specific cross‐sectional associations and should not be interpreted as evidence that disinformation perceptions preceded depressive symptoms.

### 3.2. RQ2: Is the Perceived Credibility of Individual Information Narratives Associated With GAD‐7 and PHQ‐9 Scores Among Czech Respondents?

The outcomes of the analytical processes in Tables 7 and 8 are related to RQ2.

**Table 7:** Simple quasi‐Poisson regression analysis—linking disinformation news to GAD‐7 (score) in age group classification.

Term	Aged 16–39			Aged 40–64			Aged 65–90		
	*β* (95% CI)	*p*‐Value *q*	*R* ^2^	*β* (95% CI)	*p*‐Value *q*	*R* ^2^	*β* (95% CI)	*p*‐Value *q*	*R* ^2^
*α*	1.4 (1.23–1.57)	<0.001 —	0.019|0.09	1.1 (0.952–1.25)	<0.001 —	0.008|0.042	0.588 (0.349–0.827)	<0.001 —	0.015|0.069
DEZ01A	0.182 (0.084–0.28)	<0.001 0.0027		0.136 (0.053–0.218)	0.001 0.0027		0.230 (0.082–0.323)	0.001 0.0027	
*α*	1.43 (1.27–1.59)	<0.001 —	0.018|0.083	1.08 (0.925–1.23)	<0.001 —	0.009|0.049	0.706 (0.461–0.951)	<0.001 —	0.006|0.029
DEZ01B	0.15 (0.065–0.236)	0.001 0.0027		0.129 (0.056–0.201)	<0.001 0.0027		0.128 (0.008–0.247)	0.036 0.0594	
*α*	1.43(1.24–1.61)	<0.001 —	0.014|0.065	0.877 (0.703–1.05)	<0.001 —	0.024|0.12	0.338 (0.043–0.632)	0.025 —	0.027|0.124
DEZ01C	0.127 (0.043–0.21)	0.003 0.0068		0.194 (0.126–0.263)	<0.001 0.0027		0.253 (0.141–0.365)	<0.001 0.0027	
*α*	1.43 (1.25–1.6)	<0.001 —	0.016|0.073	0.997 (0.84–1.15)	<0.001 —	0.015|0.08	0.572 (0.317–0.826)	<0.001 —	0.014|0.065
DEZ01D	0.151 (0.059–0.243)	0.001 0.0027		0.185 (0.104–0.265)	<0.001 0.0027		0.215 (0.083–0.347)	0.001 0.0027	
*α*	1.5 (1.32–1.68)	<0.001 —	0.007|0.035	1.22 (1.04–1.4)	<0.001 —	0.001|0.005	0.42 (0.117–0.723)	0.007 —	0.018|0.085
DEZ01E	0.099 (0.01–0.188)	0.030 0.0508		0.048 (−0.036–0.131)	0.264 0.3057		0.249 (0.116–0.381)	<0.001 0.0027	
*α*	1.5 (1.29–1.7)	<0.001 —	0.005|0.026	1.12 (0.93–1.3)	<0.001 —	0.004|0.022	0.607 (0.304–0.91)	<0.001 —	0.007|0.036
DEZ01F	0.094 (−0.006–0.194)	0.065 0.0998		0.101 (0.014–0.188)	0.023 0.0410		0.157 (0.025–0.289)	0.020 0.0377	
*α*	1.37 (1.18–1.55)	<0.001 —	0.02|0.094	0.992 (0.827–1.16)	<0.001 —	0.014|0.075	0.467 (0.205–0.729)	<0.001 —	0.021|0.099
DEZ01G	0.161 (0.075–0.248)	<0.001 0.0027		0.167 (0.092–0.243)	<0.001 0.0027		0.235 (0.12–0.351)	<0.001 0.0027	
*α*	1.48 (1.26–1.69)	<0.001 —	0.006|0.029	1.27 (1.08–1.45)	<0.001 —	<0.001 0.001	0.507 (0.203–0.81)	0.001 —	0.013|0.061
DEZ01H	0.097 (0–0.193)	0.050 0.0786		0.022 (−0.058–0.102)	0.586 0.6238		0.181 (0.064–0.297)	0.002 0.0051	
*α*	1.86 (1.62–2.11)	<0.001 —	0.004|0.019	1.43 (1.19–1.67)	<0.001 —	0.001|0.004	0.636 (0.177–1.1)	0.007 —	0.003|0.013
DEZ01I	−0.066 (−0.149–0.016)	0.116 0.1576		−0.038 (−0.115–0.039)	0.331 0.3767		0.092 (−0.043–0.227)	0.182 0.2224	
*α*	0.17 (1.42–1.99)	<0.001 —	<0.001	1.54 (1.32–1.77)	<0.001 —	0.003|0.018	0.719 (0.353–1.08)	<0.001 —	0.002|0.011
DEZ01J	−0.009 (−0.108–0.09)	0.856 0.8692		−0.083 (−0.163 to −0.003)	0.041 0.0660		0.076 (−0.044–0.196)	0.216 0.2592	
*α*	1.97 (1.74–2.2)	<0.001 —	0.011|0.05	1.59 (1.36–1.83)	<0.001 —	0.005|0.025	0.987 (0.532–1.44)	<0.001 —	<0.001
DEZ01K	−0.113 (−0.197 to −0.028)	0.009 0.0198		−0.097 (−0.175 to −0.019)	0.015 0.0300		−0.016 (−0.159–0.128)	0.830 0.8559	

*Note:* Unadjusted *p*‐values are reported on the first line and Benjamini–Hochberg false discovery rate (FDR)‐adjusted *q*‐values on the second line. Adjustment was applied across the 66 tests in the RQ2 family; intercepts were excluded from the multiple‐testing correction.

**Table 8:** Simple quasi‐Poisson regression analysis—linking disinformation reports with PHQ‐9 (score) in age group classification.

Term	Aged 16–39			Aged 40–64			Aged 65–90		
	*β* (95% CI)	*p*‐Value *q*	*R* ^2^	*β* (95% CI)	*p*‐Value *q*	*R* ^2^	*β* (95% CI)	*p*‐Value *q*	*R* ^2^
*α*	1.66 (1.51–1.81)	<0.001 —	0.019|0.085	1.46 (1.34–1.58)	<0.001 —	0.01|0.053	1.23 (1.07–1.39)	<0.001 —	0.008|0.031
DEZ01A	0.159 (0.072–0.245)	<0.001 0.0027		0.128 (0.061–0.195)	<0.001 0.0027		0.108 (0.023–0.193)	0.013 0.0277	
*α*	1.69 (1.55–1.83)	<0.001 —	0.017|0.075	1.5 (1.38–1.62)	<0.001 —	0.007|0.034	1.32 (1.16–1.49)	<0.001 —	0.002|0.007
DEZ01B	0.128 (0.052–0.203)	0.001 0.0027		0.09 (0.03–0.149)	0.003 0.0068		0.0504 (−0.033–0.134)	0.237 0.2793	
*α*	1.65 (1.49–1.81)	<0.001 —	0.018|0.079	1.28 (1.14–1.42)	<0.001 —	0.027|0.13	1.12 (0.922–1.31)	<0.001 —	0.013|0.053
DEZ01C	0.126 (0.053–0.198)	0.001 0.0027		0.17 (0.115–0.226)	<0.001 0.0027		0.128 (0.05–0.205)	0.001 0.0027	
*α*	1.66 (1.51–1.81)	<0.001 —	0.018|0.081	1.42 (1.29–1.55)	<0.001 —	0.014|0.069	1.17 (0.998–1.34)	<0.001 —	0.012|0.047
DEZ01D	0.143 (0.062–0.224)	0.001 0.0027		0.145 (0.079–0.211)	<0.001 0.0027		0.144 (0.052–0.236)	0.002 0.0051	
*α*	1.79 (1.64–1.95)	<0.001 —	0.003|0.015	1.54 (1.4–1.69)	<0.001 —	0.003|0.013	1.18 (0.973–1.38)	<0.001 —	0.007|0.03
DEZ01E	0.059 (−0.021–0.138)	0.147 0.1915		0.063 (−0.005–0.13)	0.068 0.1020		0.115 (0.022–0.208)	0.015 0.0300	
*α*	1.75 (1.57–1.94)	<0.001 —	0.005|0.021	1.54 (1.39–1.69)	<0.001 —	0.003|0.013	1.27 (1.07–1.48)	<0.001 —	0.003|0.011
DEZ01F	0.076 (−0.012–0.164)	0.091 0.1306		0.052 (−0.006–0.136)	0.072 0.1056		0.067 (−0.025–0.159)	0.151 0.1917	
*α*	1.61 (1.45–1.77)	<0.001 —	0.023|0.102	1.43 (1.29–1.56)	<0.001 —	0.012|0.058	1.17 (0.997–1.35)	<0.001 —	0.011|0.043
DEZ01G	0.151 (0.075–0.227)	<0.001 0.0027		0.124 (0.062–0.186)	<0.001 0.0027		0.122 (0.041–0.204)	0.003 0.0068	
*α*	1.76 (1.57–1.95)	<0.001 —	0.004|0.018	1.61 (1.46–1.76)	<0.001 —	<0.001 |0.003	1.2 (0.999–1.41)	<0.001 —	0.006|0.025
DEZ01H	0.068 (−0.017–0.153)	0.117 0.1576		0.026 (−0.038–0.091)	0.427 0.4697		0.09 (0.009–0.17)	0.029 0.0504	
*α*	1.98 (1.76–2.2)	<0.001 —	0.001|0.004	1.58 (1.38–1.78)	<0.001 —	0.001|0.003	1.21 (0.899–1.52)	<0.001 —	0.002|0.01
DEZ01I	−0.029 (−0.102–0.044)	0.441 0.4771		0.028 (−0.036–0.091)	0.393 0.4396		0.063 (−0.029–0.154)	0.180 0.2224	
*α*	1.9 (1.64–2.15)	<0.001 —	<0.001	1.87 (1.69–2.06)	<0.001 —	0.004|0.02	1.24 (0.992–1.49)	<0.001 —	0.003|0.011
DEZ01J	0.001 (−0.086–0.088)	0.986 0.9860		−0.075 (−0.14 to −0.01)	0.023 0.0410		0.06 (−0.021–0.142)	0.148 0.1915	
*α*	2.24 (1.93–2.34)	<0.001 —	0.009|0.04	1.82 (1.63–2.01)	<0.001 —	0.002|0.01	1.47 (1.16–1.78)	<0.001 —	<0.001 |0.001
DEZ01K	−0.09 (−0.164 to −0.016)	0.018 0.0349		−0.053 (−0.117–0.011)	0.107 0.1503		−0.019 (−0.117–0.079)	0.704 0.7375	

*Note:* Unadjusted *p*‐values are reported on the first line and Benjamini–Hochberg false discovery rate (FDR)‐adjusted *q*‐values on the second line. Adjustment was applied across the 66 tests in the RQ2 family; intercepts were excluded from the multiple‐testing correction.

Table 7 presents the outcomes of the simple quasi‐Poisson regression models that examined the relationship between the perceived credibility of individual information narratives (DEZ01A–DEZ01K) and GAD‐7 anxiety symptom scores, with analyses conducted separately for the three age categories. After FDR adjustment across the 66 narrative‐specific tests, 20 of the 33 GAD‐7 narrative‐by‐age associations remained statistically significant (*q* <0.05). Positive associations for DEZ01A, DEZ01C, DEZ01D, and DEZ01G remained significant in all three age groups. DEZ01B remained significant in the 16–39 and 40–64 groups but not in the 65–90 group (*q* = 0.0594). DEZ01E remained significant only in the oldest group, DEZ01F in the 40–64 and 65–90 groups, and DEZ01H only in the oldest group. The negative associations for the factual narrative DEZ01K remained significant in the 16–39 and 40–64 groups. DEZ01I showed no FDR‐retained association, and the unadjusted association for DEZ01J in the 40–64 group (*p* = 0.041) did not survive correction (*q* = 0.0660). The unadjusted DEZ01E association in the youngest group (*p* = 0.030) likewise did not remain significant (*q* = 0.0508). Thus, the corrected results retain a substantial but more selective pattern of narrative‐specific associations with anxiety symptoms.

Table 8 presents the outcomes of the simple quasi‐Poisson regression models examining associations between the perceived credibility of individual information narratives and PHQ‐9 depressive symptom scores across the three age groups. After FDR adjustment, 17 of the 33 PHQ‐9 narrative‐by‐age associations remained statistically significant (*q* <0.05). Positive associations for DEZ01A, DEZ01C, DEZ01D, and DEZ01G remained significant in all three age groups. DEZ01B remained significant in the 16–39 and 40–64 groups, DEZ01E only in the 65–90 group, and the negative association for DEZ01J remained significant only in the 40–64 group. The negative association for the factual narrative DEZ01K remained significant in the 16–39 group. No DEZ01F or DEZ01I association survived the correction. The DEZ01H association in the oldest group, which was significant before correction (*p* = 0.029), was marginally above the FDR threshold after adjustment (*q* = 0.0504). The corrected PHQ‐9 results therefore show a smaller and more selective set of narrative‐specific associations than the unadjusted analyses.

## 4. Discussion

The main goal of the study was to examine the relationship between anxiety and depressive symptoms and the perception of current disinformation from various thematic fields across narratives concerning health, social, environmental, economic, and geopolitical issues. The achievement of the study objective was supported by two RQs.

The FDR‐adjusted analyses support a more conservative version of the original pattern. Perceived exposure to disinformation remained positively associated with both GAD‐7 and PHQ‐9 scores across all age groups, while narrative‐specific associations were reduced to 20 of 33 GAD‐7 tests and 17 of 33 PHQ‐9 tests. The corrected pattern varied across narratives and age groups and did not identify a single age group as uniformly the most susceptible or most strongly associated with all outcomes. Previous studies provide context for these findings. Kitamura et al. [50], who analysed the COVID‐19 infodemic, reported associations between infodemic exposure and symptoms of depression and generalised anxiety among older people. They also discussed the potential for social‐media information environments to intensify stress, fear, depressive symptoms, and anxiety. Gong and Ren [37] highlighted the need to investigate relationships between online disinformation, PTSD‐related psychological mechanisms, and social‐media exposure among earthquake survivors. Dukuye [51] similarly suggested that pandemic‐related anxiety and depression may have been aggravated by exposure to fake news and that cognitive reflection was associated with the ability to discriminate between true and false headlines. Prisă [17] identified age‐related differences in negative emotional states associated with fake‐news exposure. Hudon et al. [52] further argued that disease‐related disinformation may be particularly harmful for vulnerable individuals by increasing isolation and helplessness. Importantly, the present findings are cross‐sectional associations and do not demonstrate that perceived disinformation caused anxiety or depressive symptoms.

There are three explanations compatible with the findings at least. Firstly, perceived exposure to disinformation may function as an informational stressor by increasing uncertainty, perceived threat, information overload, and repetitive searching. Secondly, reverse directionality is plausible: anxiety may heighten threat vigilance and sensitivity to alarming information, while depressive symptoms may be associated with rumination and negative interpretative bias, which could influence perceived exposure or credibility. Thirdly, shared factors such as sex, education, socioeconomic status, employment, media use, institutional trust, previous mental‐health difficulties, and exposure to stressful events may affect both disinformation perceptions and symptom scores.

Taken together, these pathways form a bidirectional informational‐stress and cognitive‐vulnerability framework. Within this framework, a disinformation‐saturated environment may operate as a perceived informational stressor by increasing uncertainty, threat vigilance, information overload, and repetitive information seeking. At the same time, anxiety and depressive symptoms may shape how individuals notice, interpret, and evaluate information through heightened threat sensitivity, rumination, and negative interpretative bias. The observed associations may also arise partly from shared social, cognitive, and psychological vulnerabilities. This framework therefore does not assume a single causal direction; instead, it treats perceived disinformation and mental‐health symptoms as potentially reciprocal processes embedded in a broader information environment.

Based on the outcomes of the GAD‐7 and PHQ‐9 scales, the descriptive results show that a substantial part of the Czech population reported mild to moderate anxiety or depressive symptoms. Higher symptom scores were associated with more intense perceived exposure to disinformation (IP02) and in some age groups with perceiving disinformation as a serious problem (IP04). Associations between symptom scores and the credibility of individual disinformation narratives were also evident. The direction of these relationships remains uncertain: symptoms may shape perceptions, perceptions may contribute to symptoms, or both may reflect shared vulnerabilities. For instance, Prisă [17] showed that psychological vulnerabilities, including an irrational cognitive profile, externalism, and intolerance of uncertainty, explained a substantial proportion of negative emotional responses associated with fake news. Khalaf and Shehata [12] reported that exposure to personal contacts and digital media was associated with stronger disinformation and conspiracy beliefs. The thematic content of a narrative may also matter because individuals may interpret health, geopolitical, economic, environmental, or social claims differently depending on their perceived implications for personal safety, future prospects, and social or economic security.

GAD‐7 retained somewhat more narrative‐specific associations after FDR correction than PHQ‐9 (20 vs. 17 of 33 tests), supporting the interpretation that anxiety‐related associations were modestly more consistent. However, the difference was not large, and the corrected PHQ‐9 associations were not concentrated exclusively among younger respondents. The results therefore support consideration of both anxiety and depressive symptoms without implying a uniform age‐specific vulnerability. Analytical thinking offers one possible mechanism for linking the information environment with these outcomes. An important theoretical question is whether anxiety, distraction, or cognitive overload reduces the use of reflective, accuracy‐oriented thinking and thereby increases vulnerability to false claims. Kwek et al. [38] suggest that psychological factors can distract from analytical evaluation and that analytical thinking protects against disinformation primarily when it is directed towards assessing truth. Conversely, accuracy‐focused analytical thinking may reduce uncertainty by supporting source checking and evidence evaluation. Analytical thinking was not measured in this study and should therefore be examined as a potential mediator or moderator rather than be treated as an established explanation of the present findings.

The possible link between analytical thinking and anxiety is therefore likely to be conditional rather than linear. Anxiety may motivate careful checking when a person has sufficient cognitive resources, time, confidence, and media‐literacy skills. However, higher anxiety may also narrow attention towards threat, increase urgency, encourage repetitive searching, and impair reflective evaluation. Analytical thinking may consequently buffer the association between perceived disinformation and distress under some conditions, while cognitive overload, intolerance of uncertainty, or rumination may weaken this protection. Future studies should measure analytical thinking, intolerance of uncertainty, rumination, information overload, and media literacy alongside GAD‐7 and PHQ‐9 to test these competing pathways directly.

Age differences remained important descriptively, but the FDR‐adjusted results do not support a simple age gradient in the associations between disinformation‐related perceptions and mental‐health symptoms. Respondents aged 16–39 had the highest mean GAD‐7 and PHQ‐9 scores. However, after FDR correction, the number of the 11 information narratives showing statistically significant associations with GAD‐7 was similar across age groups: 6 narratives among respondents aged 16–39, 7 among those aged 40–64, and 7 among those aged 65–90. For PHQ‐9, significant associations were retained for 6, 6, and 5 narratives in the three age groups, respectively. In the models examining general perceptions of disinformation, perceived exposure remained positively associated with GAD‐7 in all three age groups, with the largest coefficient observed in the 65–90 group, whereas perceived seriousness of disinformation showed the most consistent pattern in the 40–64 group, where it remained associated with both GAD‐7 and PHQ‐9. Taken together, these findings indicate age‐related heterogeneity in the pattern of associations rather than a uniform ordering of vulnerability across age groups.

Age‐related differences may also intersect with gender, education, media use, and social position, but the evidence is mixed; for example, Khalaf and Shehata [12] found no gender differences in disinformation beliefs. Crisis‐related information distributed through social media may increase confusion, perceived threat, and public distrust, while repeated exposure may also be associated with poorer mental‐health outcomes [53, 54]. Disinformation and conspiracy theories may additionally undermine trust in healthcare, exacerbate health inequalities, and contribute to harmful health behaviours among marginalised populations [55]. These explanations remain tentative because social status, gender, education, media use, and sensitivity to information threats were not tested as explanatory mechanisms in the age‐stratified models.

Broader media‐system and political characteristics may shape both exposure to disinformation and its mental‐health relevance. Political polarisation, populist communication, the strength of public‐service media, audience fragmentation, social‐media use, and the structure of digital advertising markets may influence the amount and type of disinformation encountered [18]. Arechar et al. [56] found that several psychological correlates of disinformation attitudes recur across regional settings, suggesting that some preventive principles may be transferable. Nevertheless, contextual factors should be treated as possible moderators rather than direct explanations of the present results because they were not measured in this study. Future comparative research should examine whether institutional trust, media‐system characteristics, and political context alter the associations between perceived disinformation and anxiety or depressive symptoms.

Information overload provides a plausible mechanism connecting disinformation environments with mental‐health symptoms. High volumes of rapidly changing or conflicting information may increase uncertainty and stress [57–60], while simultaneously reducing the motivation and cognitive capacity required for source verification. Time spent online and intensive social‐media use have also been associated with exposure to and dissemination of false information [61, 62]. Individuals who repeatedly search for crisis‐related information, may enter a reinforcing cycle in which uncertainty encourages further information seeking; therefore, increased exposure intensifies distress, and distress makes reflective evaluation more difficult [63]. This mechanism remains hypothetical in the present study because information overload, repetitive searching, and time spent on social media were not measured directly.

The implications should distinguish interventions addressing disinformation from interventions addressing anxiety and depressive symptoms. Information‐integrity programmes may include source‐verification training, lateral reading, accuracy prompts, recognition of emotional manipulation, and practical strategies for managing information overload [64]. These programmes could be delivered through schools, community organisations, public‐service media, libraries, fact‐checking organisations, and targeted digital‐literacy initiatives for vulnerable age groups [65]. Mental‐health prevention and care should separately include psychoeducation, coping and stress‐management skills, guidance on limiting compulsive crisis‐information consumption, referral pathways, and access to evidence‐based psychological or psychiatric care when symptoms are clinically significant [66]. Media‐literacy education should not be presented as a treatment for anxiety or depression [67]. The two approaches are complementary and require collaboration among educators, fact‐checkers, public‐health professionals, clinicians, media organisations, and public institutions. Their effectiveness should be evaluated separately using outcomes appropriate to information resilience and mental health [68].

The practical implications of these findings should be differentiated according to the principal objective of the intervention. Programmes targeting misinformation should primarily strengthen media and information literacy through training in source evaluation, lateral reading, fact‐checking, recognition of emotionally manipulative content, and reflection before sharing unverified information. Such activities could be implemented through schools, universities, community organisations, public information campaigns, libraries, healthcare institutions, and digital platforms, with content adapted to the needs of different age and risk groups. By contrast, programmes aimed at preventing or reducing psychological distress should focus on stress management, emotional regulation, healthy patterns of social media use, access to psychological support, and referral to appropriate professional services when necessary. Although these two types of programmes have distinct objectives, they may be coordinated within integrated preventive initiatives. For example, media literacy training could include guidance on managing emotional responses to alarming online content, while mental health programmes could address the psychological effects of repeated exposure to misleading or threatening information. However, misinformation education should not be presented as a substitute for mental health prevention or treatment, and the effectiveness of such combined interventions should be evaluated in longitudinal or experimental studies.

These findings should nevertheless be interpreted with caution because the cross‐sectional design does not allow the direction of the observed associations to be established. Although exposure to and engagement with misinformation may contribute to psychological distress, reverse causality is also plausible: individuals experiencing higher levels of depression or anxiety may be more susceptible to misleading information, more likely to interpret online content negatively, or more inclined to engage with emotionally charged narratives [40, 69]. Moreover, both misinformation‐related behaviours and psychological distress may be influenced by shared sociodemographic, socioeconomic, educational, political, or contextual factors that were not fully captured in the present analysis. The results therefore indicate associations rather than causal effects. Future research should employ longitudinal, experimental, or panel designs to examine temporal ordering, reciprocal relationships, and the mechanisms through which disinformation exposure and mental health may influence one another.

## 5. Conclusion

This study identified cross‐sectional associations between perceived exposure to disinformation, perceived seriousness of online disinformation, the credibility of selected information narratives, and anxiety and depressive symptom scores in the Czech population. After FDR correction, perceived exposure remained associated with both outcomes across all age groups, while 20 of 33 GAD‐7 and 17 of 33 PHQ‐9 narrative‐specific associations remained statistically significant. The corrected results indicate heterogeneous narrative‐ and age‐specific patterns rather than a uniform age gradient. However, the findings do not establish that disinformation causes anxiety or depressive symptoms. The relationship may be reciprocal: perceived disinformation may contribute to uncertainty and information‐related stress, while anxiety and depressive symptoms may increase threat sensitivity, rumination, and negative interpretation of information. Both domains may also be influenced by shared social, cognitive, and psychological factors. The study contributes by proposing a bidirectional informational‐stress and cognitive‐vulnerability framework and by extending research beyond pandemic‐specific misinformation to diverse contemporary narratives. Longitudinal and experimental research should directly test temporal direction and examine analytical thinking, intolerance of uncertainty, rumination, information overload, and media literacy as mediators or moderators. Prevention should combine information‐integrity interventions with separate, evidence‐based mental‐health support rather than treating media literacy as a substitute for clinical care.

## 6. Limitations

The principal limitation is the cross‐sectional design, which prevents conclusions about temporal order and causality. Anxiety or depressive symptoms may influence perceived exposure, seriousness, or credibility; perceived disinformation may contribute to symptoms, and shared factors may influence both. The analyses did not adjust for additional individual sociodemographic characteristics such as sex, educational attainment, employment status, or place of residence, so unmeasured confounding remains possible. The CAWI panel‐based design also introduces potential selection bias. The final analytical sample represented 68.3% of invited panel members, but the excluded pool combined nonrespondents, incomplete questionnaires, attention‐check exclusions, and records removed during data quality control. Comparable demographic information was not available for all of these groups, preventing a valid direct comparison of the 2867 analysed respondents with the entire excluded pool. Quota sampling reduces but does not eliminate residual nonresponse and panel‐selection bias.

A further limitation concerns the interpretation and contextual validity of the narrative‐specific measures. The 11 credibility items were not psychometrically validated as a unified measure of general susceptibility to disinformation. Knowing or judging whether a brief claim is credible may reflect topic‐specific knowledge, prior exposure, political or social attitudes, familiarity, and other predispositions. In addition, the factual meaning and perceived credibility of information depend partly on source cues, surrounding discourse, expert references, claims to objectivity, and the broader context in which a claim is encountered [70]. Because respondents evaluated brief statements without their original source or presentation context, the study could not assess source evaluation, lateral reading, contextual fact‐checking, or recognition of manipulative framing. Several narratives were also specifically related to Czech political, social, economic, or institutional circumstances; narrative‐specific results should therefore not be generalised directly to other national settings or interpreted as a stable general trait of disinformation susceptibility.

Finally, the large number of comparisons increased the risk of Type I error. This revision addresses that concern by applying Benjamini–Hochberg FDR adjustment to the 15 preliminary Kruskal–Wallis comparisons as a separate screening family and within the prespecified RQ1 and RQ2 regression‐test families, with statistical interpretation based on *q* <0.05. The correction reduced the number of narrative‐specific findings considered statistically significant and supports a more cautious interpretation of the remaining associations. The self‐reported exposure measure may also capture perceived rather than objective contact with disinformation. Future longitudinal, experimental, and cross‐national research should test directionality, contextualised information evaluation, analytical thinking, and the roles of sociodemographic and media‐related confounders.

## Author Contributions

**Vaclav Moravec**: conceptualisation, methodology, writing – original draft, writing – review and editing, visualisation, validation, supervision. **Beata Gavurova**: formal analysis, methodology, data curation, writing – original draft, writing – review and editing, visualisation. **Martin Rigelsky**: formal analysis, data curation, methodology, software, validation. **Michal Miovsky**: conceptualisation, writing – original draft, writing – review and editing, supervision.

## Funding

The paper was financed by the project “Support for increasing the impact, innovation and sustainability of CEDMO” in the Czech Republic (Grant 1.4 CEDMO 1 ‐ Z220312000000). This project is funded by the EU’s Recovery and Resilience Facility (RRF) and institutional support from Charles University (Program Cooperatio: Health Sciences).

## Ethics Statement

All participants were informed about the study procedures and provided written informed consent prior to participation. The study was conducted in accordance with the Declaration of Helsinki and was approved by the Ethics Committee of the General University Hospital in Prague (IORG0002175—General University Hospital in Prague, IRB00002705, and Federalwide Assurance FWA00029052).

## Conflicts of Interest

The authors declare no conflicts of interest.

## Data Availability

The individual‐level research data are not publicly available because the participant information and ethical approval did not explicitly provide for unrestricted public dissemination of the research dataset. The analyses were conducted on anonymized data. Any future access to individual‐level data would require compliance with applicable institutional, ethical, and data‐protection requirements.
